# Large scale variation in DNA copy number in chicken breeds

**DOI:** 10.1186/1471-2164-14-398

**Published:** 2013-06-13

**Authors:** Richard PMA Crooijmans, Mark S Fife, Tomas W Fitzgerald, Shurnevia Strickland, Hans H Cheng, Pete Kaiser, Richard Redon, Martien AM Groenen

**Affiliations:** 1 Animal Breeding and Genomics Centre, Wageningen University, P.O. box 338, Wageningen 6700 AH, The Netherlands; 2 Genetics and Genomics group, Compton Laboratory, The Pirbright Institute, Compton, Berkshire RG20 7NN, UK; 3 Wellcome Trust Genome Campus, Wellcome Trust Sanger Institute, Hinxton, Cambridge CB10 1SA, UK; 4 L'institut du thorax, UMR Inserm 1087 CNRS 6291, University of Nantes, 8 quai Moncousu, BP 70721, Nantes, Cedex 1 44007, France; 5 Department of Animal & Food Sciences, University of Delaware, Newark, DE 19717, USA; 6 Avian Disease and Oncology Laboratory, 4279 E Mount Hope Road, East Lansing, MI 48823-5338, USA; 7 The Roslin Institute and R(D)SVS, University of Edinburgh, Easter Bush, Midlothian EH25 9RG, UK

**Keywords:** Copy number variation, Chicken, aCGH, Line-specific CNVs, Inter-specific CNVs, Genes

## Abstract

**Background:**

Detecting genetic variation is a critical step in elucidating the molecular mechanisms underlying phenotypic diversity. Until recently, such detection has mostly focused on single nucleotide polymorphisms (SNPs) because of the ease in screening complete genomes. Another type of variant, copy number variation (CNV), is emerging as a significant contributor to phenotypic variation in many species. Here we describe a genome-wide CNV study using array comparative genomic hybridization (aCGH) in a wide variety of chicken breeds.

**Results:**

We identified 3,154 CNVs, grouped into 1,556 CNV regions (CNVRs). Thirty percent of the CNVs were detected in at least 2 individuals. The average size of the CNVs detected was 46.3 kb with the largest CNV, located on GGAZ, being 4.3 Mb. Approximately 75% of the CNVs are copy number losses relatively to the Red Jungle Fowl reference genome. The genome coverage of CNVRs in this study is 60 Mb, which represents almost 5.4% of the chicken genome. In particular large gene families such as the keratin gene family and the MHC show extensive CNV.

**Conclusions:**

A relative large group of the CNVs are line-specific, several of which were previously shown to be related to the causative mutation for a number of phenotypic variants. The chance that inter-specific CNVs fall into CNVRs detected in chicken is related to the evolutionary distance between the species. Our results provide a valuable resource for the study of genetic and phenotypic variation in this phenotypically diverse species.

## Background

The chicken was the first livestock species to have its genome completely sequenced [1]: a large collection of chicken single nucleotide polymorphisms (SNPs) has been available for almost a decade [2]. More recently, the number of SNPs has been enlarged to over 7 million [3]. Although numerous studies studying genetic variation have focused on SNPs, there is growing evidence for the substantial role of structural polymorphism in phenotypic diversity [4]. Structural variation has been recognized as an important mediator of gene and genome evolution within populations [5]. While the sizes of genetic variants range from a single base to whole chromosomes, historically only the extreme ends of the spectrum have been explored. DNA copy number variants (CNVs) lie between these two extremes, ranging in size from thousands to millions of bases.

In human, many CNVs are in linkage disequilibrium with nearby genetic markers and thus appear to be ancient [6]. Others are more recent, such as CNVs affecting olfactory receptor gene diversity [7], or can be recurrent [8]. Structural variants include a variety of molecular alterations such as duplications, deletions, and inversions [9,10]. A comprehensive map that catalogues and indexes structural variants - in particular CNVs - across the genome is a necessary prelude to understanding their role in the context of specific phenotypic traits. Early reports estimated that at least 2% of the human genome is affected by structural variations [11], but more recent studies suggest that as much as 3.75% of the human genome harbors common CNVs [12].

CNV regions (CNVRs) will be an important complement to SNP-centric genome-wide association studies, since existing SNP discovery and genotyping methodologies are biased against inclusion of these more complex genetic variants. Furthermore, many of the CNVRs are not very well represented and annotated in the genomic sequence due to biases in chromosome assembly. In order to estimate what fraction of the genome is affected by CNV, global studies have been performed in human, chimpanzee, dog, mouse and cattle. In cattle, for example, 177 high confidence CNVRs were reported as covering 28.1 Mb, 35 of these CNVRs being apparently breed-differential or breed-specific [13]. To determine the full extent of variation and its influence on phenotypic variation, the reference genome assembly should be near completion and more individual genomes need to be sequenced for the species of interest. Analysis of CNVs in livestock species is of particular interest, not only because of their economic importance, but also due to the often- extensive selection pressure applied in generating the different lines and varieties.

Since CNVs potentially affect gene expression [8], CNVs may account for a significant proportion of the extensive phenotypic variation observed in this species. Examples of phenotypes associated with a CNV in the chicken include late feathering on chromosome Z (GGAZ) [14], pea comb on GGA1 [15], dark brown plumage color on GGA1 [16] and dermal hyperpigmentation on GGA20 [17]. Additional CNVs have been detected in the chicken using aCGH [18], but that study only examined ten individuals and only identified 96 CNVs corresponding to approximately 1.3% of the chicken genome. Furthermore only 27 of these CNVs were observed in more than one individual.

Here we applied an aCGH analysis to different chicken breeds in order to obtain a global CNV map of the chicken genome.

## Results and discussion

### CNV in chicken

aCGH was carried out using the Agilent 244K chicken array with a mean probe spacing of 4000 bp. This array is based on the chicken assembly WUSTL 2.1 (Galgal3) and covers chromosomes 1–28, 32 and the sex chromosomes Z and W. The virtual chromosome “ChrUn” with concatenated unmapped contigs was not taken into account in the probe design. To access the chicken CNV landscape, we selected 64 animals from 6 commercial lines (layer and broiler types), 7 experimental lines (layer and broiler types), Red Jungle Fowls and Silkies. DNA samples were labeled with Cy3 whereas the reference DNA sample - derived from UCD001, the Red Jungle Fowl animal previously selected to generate the chicken reference genome assembly - was labeled with Cy5.

We defined conservative parameters for CNV detection to limit false positive calls (see Methods). Within the 15 lines used in this study, 3,154 CNVs with a different start and/or end location on the chicken genome were detected (Additional file 1). Seventy-five percent of these CNVs are losses. Being more conservative, i.e. requiring the CNV to be observed in at least 2 samples, 944 CNVs (29.9%) were detected with an average size of 46.1 kb. The real time PCR validation of 12 CNVs ranging in being present in 1 to 41 samples did give a successful validation of 92%. Only one marker in a potential CNV which was detected in only one animal failed validation by showing no difference between the reference sample. These results indicated that the detected CNVs have a high chance of being a real CNVs. Furthermore, we could confirm 13 of the 26 high confidence CNVs (50%) identified by Wang et al. (2010) and 23 out of the 70 CNVs (23.9%) detected in only one animal in that study. However, we were only able to confirm 21% of the 238 CNVs detected in another study (Wang et al. 2012) (Additional file 1). One reason why fewer CNVs were detected in the studies of Wang et al. [18,19] is the use of only 10 and 6 animals respectively from three different breeds in those two studies. Moreover, both studies used a different reference animal and the reference animal was also from the same breed. Also, none of the chicken breeds were in common between our study and those of Wang [18,19] which may account for the lack of complete validation.

**Figure 1:**
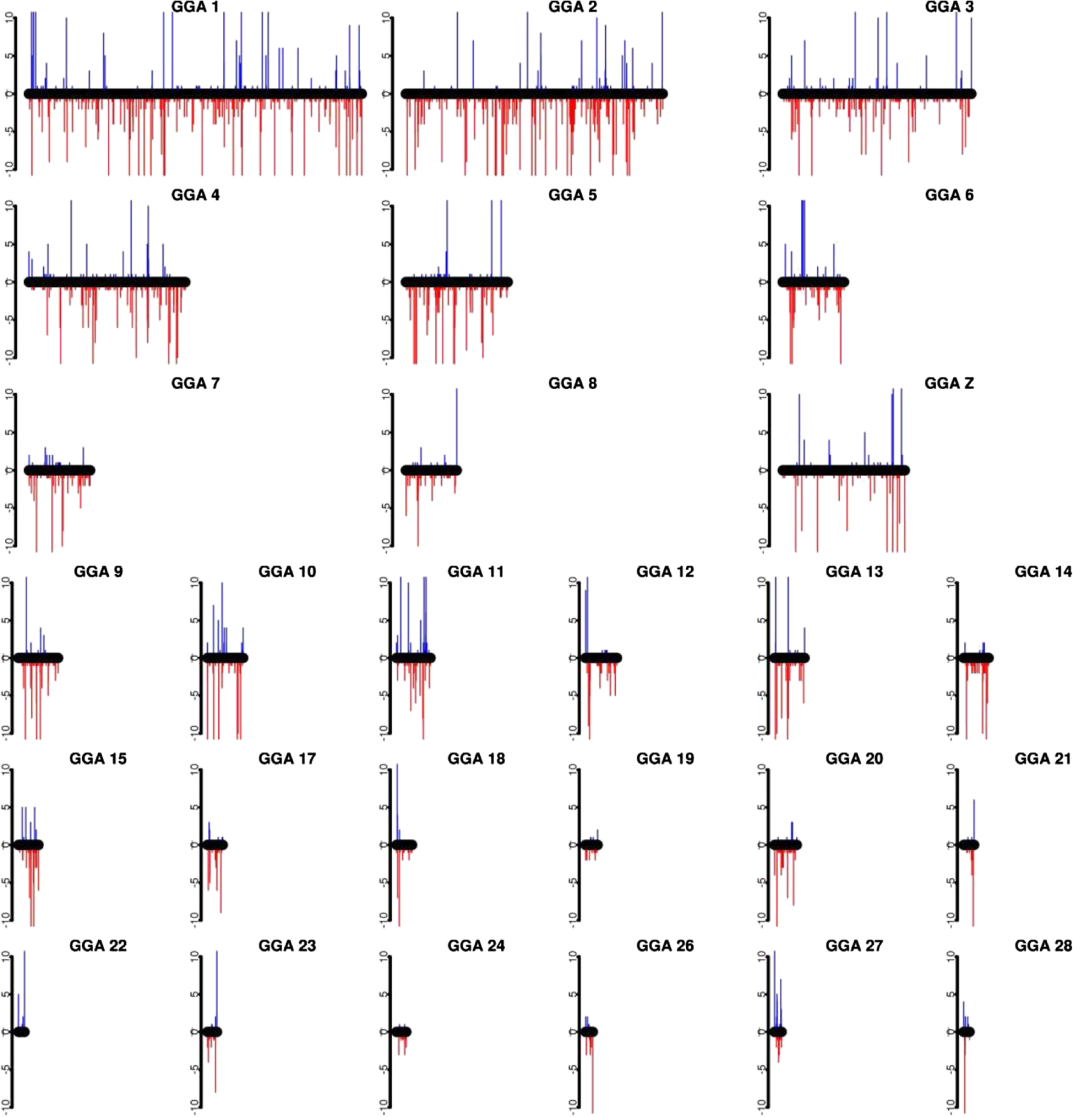
**CNV distribution across the chicken genome.** Red bars indicate copy-number losses and blue bars copy-number gains. Bar length indicates the number of occurrence for a given CNV, divided into four groups: one contains ≥ 10 animals carrying this CNV; the second is ≥ 5 and <10 animals; the third is ≥ 2 and <5 and the last group has only one animal carrying this CNV.

The distribution of the gain and loss CNVs over the genome is shown in Figure 1. The average size of the CNVs detected in this study is 46.3 kb, and the largest CNV, 4.3 Mb (CNV #3126), was observed on GGAZ. CNV distributions within the different chicken lines are given in Additional file 1: Table S1. Although limited sequence information is available for GGA16 (MHC-containing chromosome), the repetitive nature of this chromosome [1] was confirmed by detecting copy number variations on the entire sequenced part of this chromosome. Another striking case was seen on GGA25 where 16 out of the 64 animals showed CNVs in a CNVR covering a substantial part of the genome sequence (from 0 up to 1.85 Mb). Interestingly, GGA25 is one of the more GC-rich chromosomes and it contains a relatively large number of minisatellites [20].

**Figure 2:**
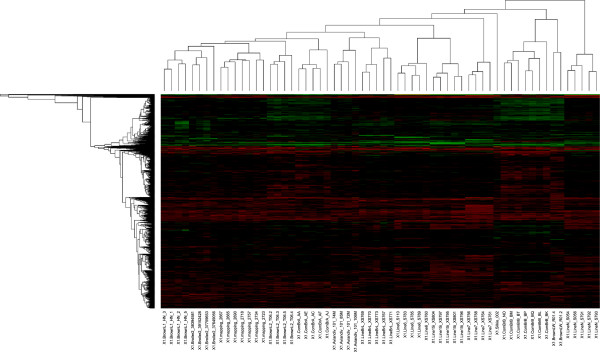
**Heatmap representation of copy number variation between chickens.** Unsupervised clustering of CNVs with gains (green) and losses (red) yields a dendrogram that recapitulates features of the known genealogy of these animals within a line or breed.

The average number of CNVs per animal was 103, ranging from 61 to 209. The highest number of CNVs was detected in the commercial White Leghorn line with an average of 187.5 CNV per animal. The lowest number of CNVs was observed in the Red Jungle Fowl (Aviandiv) population with an average of 83.8 CNV per animal, which was expected as an inbred Red Jungle Fowl animal was used as a reference in this study. The commercial broilers showed an average of 128.8 CNVs per animal. These numbers are considerably higher than those reported by Wang et al. (2012), where the average was 40 CNVs per animal. Even when our analysis is restricted to the autosomes, as was done in the study of Wang et al. (2012), we still observe many more CNVs (118.8) per individual. A cluster analysis of the samples based on the CNVs detected in each of the animals results in tight clustering of all individuals from the same line (Figure 2).

**Figure 3:**
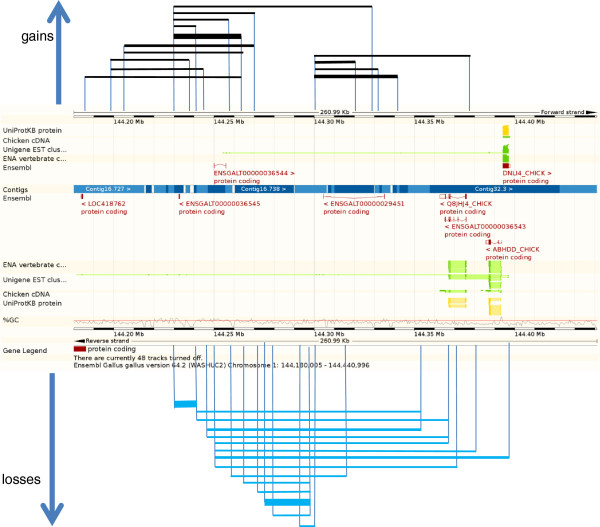
**Overview of CNVR 209 at GGA1: 144,185,960- 144,403,060.** CNVR209 (217.1 kb) consists of 30 individual CNVs (see Additional file 2: Table S2) ranging from 8.8kb to 153.7kb in size across all samples. The frequency of the CNVs within this CNVR varied from 1 to 10 across the study population. Line thickness represents the number of occurrences of the CNV. Gains are indicated with black lines, losses with blue lines.

CNVRs were determined by aggregating overlapping CNVs identified in all samples across the aCGH experiments according to the criteria defined by Redon et al. (2006). Aggregating CNVs into CNVRs resulted in a total of 1,556 non-overlapping regions covering 60 Mb, which represent almost 5.4% of the chicken genome. An example of a CNVR in the chicken is given in Figure 3. The largest CVNR detected is located on GGAZ (CNVR 1482) and is 4.37 Mb in size. The number of CNVRs in the chicken is considerably higher than that reported by Wang et al. (2010) and Wang et al. (2012), 97 and 130 respectively. The number of CNVRs in the chicken is comparable to that reported in human [21] and almost 3 times higher than in cattle [22].

**Figure 4:**
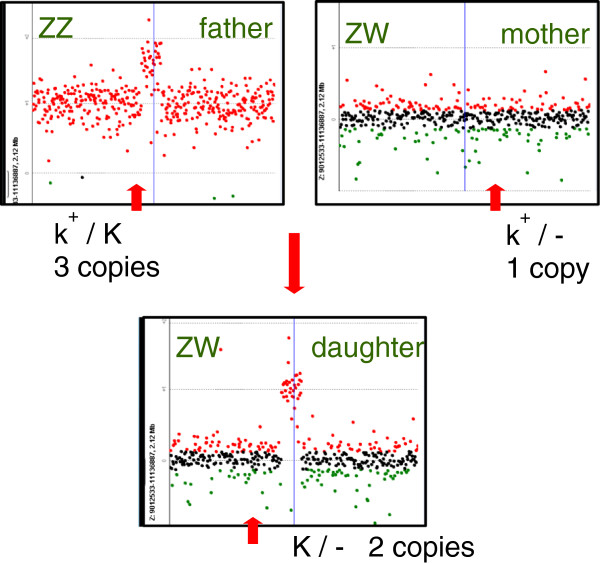
**Segregation of CNVR1508 at GGAZ: 9,971,185-10,140,048.** A male (ZZ) with k+/K (3 copies) was crossed with a female k+/− (1 copy) giving a female offspring K/- (2 copies).

The 176- kb CNV linked to the late feathering locus [14] was detected in this dataset as CNVR 1508 on GGAZ between positions 9,971,185 and 10,140,048 with a size of 169 kb. The segregation of this CNV is shown in Figure 4. As expected, we were unable to identify the CNV in intron 1 of the SOX5 gene responsible for the pea-comb phenotype in chicken due to the small size of this CNV (3.2 kb), which is below the probe spacing on our array.

### Line-specific CNVRs

Some CNVs were observed only in a single animal whereas others seem to be fixed in all individuals of one specific line. CNVs that are specific for a line or group of lines are of particular interest because these are potential candidates for genes that affect a phenotype specific for that (group of) lines. We therefore identified those CNVs that were either fixed in at least one line (defined as fixed) or that were fixed in only a single line or breed (defined as line-specific).

Within the 15 different lines used in this study, we identified 518 CNVs, comprising a total of 214 CNVRs, which were line-specific (Additional file 2). The number of CNVRs fixed within a line ranged from 2 in the Broiler Mapping population to 68 in the commercial White Layer. The number of line-specific CNVRs varied from 0 for the Broiler Mapping populations to 30 for the commercial White Layer line. The commercial White Leghorn line was only represented by two individuals, therefore resulting in a higher number of fixed CNVRs (68), of which 30 were line-specific. Of the fixed CNVRs, the number of line-specific CNVRs was high in the experimental lines and the Silkie breed (10 each).

The Silkie breed has a number of striking phenotypic characteristics such as black skin, white feathers and black bones. We therefore investigated whether there is a relation between some of these CNVRs and some of these specific phenotypes. For the Silkies, 27 fixed CNVRs were detected of which 10 were breed-specific (Additional file 2).

Two significant CNVRs are located on GGA20 (CNVR 812 at positions 10,722,231 to 10,844,289 and CNVR 814 at positions 11,263,937 to 11,435,137) and have already been described in detail by Dorshorst et al. (2011) after fine mapping of the phenotype fibromelanosis (FM) in Silkies. The candidate gene involved in pigmentation, the Endothelin 3 gene (*NDN3*), is located within CNVR 812 [17] and, when up-regulated, is the primary driver of dermal hyperpigmentation in FM chickens. The potential function of the other eight line-specific CNVRs in Silkies is not clear. One of the three Silkie line-specific CNVR on GGA27 (CNVR 889 at positions 4,128,916 to 4,155193) harbours the gene *CCR7*, which stimulates melanoma migration, and the gene *SMARCA4*, a SWI/SNF-related matrix-associated actin-dependent regulator of chromatin. Both these genes are potential candidates for traits related to pigmentation. Further studies are needed to study the full potential of these line-specific CNVRs.

For lines 6_1_ and 6_3_, we detected 29 and 36 CNVRs respectively, which are fixed in these lines, while only a single CNV and 3 CNVRs respectively are line-specific. One of the line-fixed CNVRs for line 6 (CNVR 209 on GGA1 between positions 144,249,310 and 144, 403,060) contains the gene *TNFSF13B* (tumor necrosis factor (ligand) superfamily, member 13b) or *BAFF* (B cell activation factor). This candidate gene stimulates B cells to undergo proliferation and to counter apoptosis and was examined in more detail. To confirm this CNVR, we quantified the relative abundance of the DNA copy number for *TNFSF13B* using a TaqMan assay. Quantification of the ovotranferrin gene, known to be in single copy in the chicken genome, was used as an internal reference. A primer probe set spanning exon 5 and intron 6 of *TNFSF13B* revealed a significant difference in copy number between line 6_1_ when compared to lines 7 and N. No difference was detected using a primer probe set spanning intron 1 and exon 1. These results suggest that there is partial duplication of *TNFR13B*, with exon 5 duplicated in all lines tested except in lines 6_1_ and 6_3_. However, the CNV does not extend as far as exon 1 of TNFSF13B, as indicated by the equivalent copy number across all lines at this region of the gene. Further characterisation of this CNV will be required to identify its boundaries accurately.

One of the three other line-specific CNVRs (CNVR521), detected in lines 6_1_ and 6_3,_ and located at GGA18: 372390–400489, overlaps with the Myosin Heavy Chain gene 1 (*MYH1*).

### Comparative CNV analysis

We compared the chicken CNVRs to those previously detected in turkey, duck and zebra finch (Additional file 1) [23-25]. From the 16 inter-specific CNVs detected between turkey and chicken detected by Griffin et al. (2008) using comparative CGH, 10 did not show variation in the chicken. Within the current study, 15 of these 16 inter-specific CNVs could be verified, whereas the inter-specific CNV on chrE64 could not be validated because chrE64 was not used for probe design on the array. From the 15 inter-specific regions detected, all (100%) fall into CNVRs detected in this study. The CNV detected in the chicken Layer vs. Red Jungle Fowl on GGA2 (position 25,725,000 to 25,785,000) by Griffin et al. (2008) could not be confirmed in our study. When comparing the inter-specific zebra finch / chicken CNVs [25], 9 of the 27 CNVs (33%) did overlap with a chicken CNVR while of the inter-specific CNVs detected between duck and chicken [24], 15 of the 31 (48%) did overlap with a chicken CNVR. These results indicate that the inter-specific CNVs detected are prone to overlap with a CNVR of the avian lineage when more samples are analyzed. Fewer CNVRs would be overlapping with inter-specific CNVs between more distant species.

### Gene content of chicken CNVR

Within the 1,556 CNVRs, a total of 2,642 unique Ensembl peptides were identified based on chicken build 2.1. To examine whether genes involved in specific pathways or biological processes are more prone to copy number variation, we performed a gene enrichment analysis for the genes located within the CNVRs. The chicken transcript ids were used as input into DAVID for a gene enrichment and ontology analysis [26]. Terms showing significant enrichments were the GO terms “functional constituent of cytoskeleton”, “nuclear binding”, “cellular response to stress”, and “macromolecule catabolic processes”. The GO term “functional constituent of cytoskeleton” is mainly driven by the keratin superfamily. The avian keratin genes are over-represented when compared to mammals [1]. Phylogenetic analysis demonstrated that evolution of archosaurian epidermal appendages in the linage leading to birds was accompanied by duplication and divergence of the ancestral ß-keratin gene cluster. In the chicken, four subfamilies (claw, feather, feather-like and scale) of the ß-keratin genes have been named in accordance with tissue-specific expression and sequence heterogeneity [27]. These ß-keratin gene subfamilies are clustered on GGA25 whereas the genes for two other monophyletic groups of feather keratins are located on GGA27 and GGA2 respectively. We observed large CNVRs (CNVRs 863, 873 and 791) within all three regions in the chicken genome up to 2 Mb in size. Within these CNVRs we observed both CNV losses and gains.

## Conclusions

In this study we performed aCGH screening of the chicken genome to identify CNVs in a comprehensive manner. We have identified a large number of genes affected by CNV, including genes involved in well-known phenotypes such as late feathering and pigmentation in Silkies. In particular large gene families such as the keratin gene family and the MHC show extensive variation in copy number. The CNVs in the chicken overlapping with the inter-specific CNVs (CNVs detected between different bird species) are potentially old CNVs. Moreover, when the evolutionary distance between chicken and the other bird species is enlarged the older (more ancient) the CNV is. Many of these CNVs very likely affect traits of economic importance in the chicken and our global characterization of CNVRs in the chicken genome will aid in the identification of structural variation in the genome underlying important phenotype differences for qualitative and quantitative traits.

## Methods

### Construction of the oligonucleotide microarray

A CGH array for whole genome analysis in chicken (UCSC galGal3 (WUSTL build 2.1, may 20006)) was designed and constructed by Agilent Technologies (http://www.genomics.agilent.com). The chicken genome CGH microarray kit 244A had a median probe spacing of 4 kb, with probes printed using the Agilent 60-mer Sure print technology.

### Experimental chicken lines

The experimental lines 6 (line 6_1_ and 6_3_), 7_2_ and 15I_5_ are all experimental White Leghorn lines characterized for resistance to viral-induced tumours. Line 6 was been selected for resistance to Marek’s disease (MD) and lymphoid leukosis (LL) whereas line 7_2_ is susceptible to MD and 15I_5_ is susceptible to MD and LL [28]. Lines 6_3_, 7_2_, and 15I_5_ are kept at the Avian Disease and Oncology Laboratory (ADOL) at East Lansing, MI, USA, while lines 6_1_ and 7_2_ are bred at the Pirbright Institute, Compton, UK. Line N is a control line. Line BrL is a Brown Leghorn line selected for resistance to infectious bursal disease virus (IBDV) [29-31]. All lines have been maintained by random mating within the flocks.

### Sample processing

Breeds in this study include Red Jungle Fowl, one commercial white and two commercial brown layer lines, six experimental lines (five white and one brown) three commercial broiler and one experimental broiler line, and the Silkie breed. In total 64 animals were used (Table 1). Genomic DNA was isolated from blood with the Puregene blood kit or the Qiagen Qiamap DNA blood kit. Blood samples were collected by veterinarians according to national legislation. No approval from the ethics committee was necessary according to local legislation. For the experimental lines from the Institute for Animal Health at Compton, genomic DNA was extracted from whole blood as previously described [32]. We assessed the DNA quality and quantity by OD_260/280_ and OD_260/230_ readings and on 1% agarose gels. The reference sample (UCD001) used is the same individual that was used to generate the chicken genome reference sequence [1].

**Table 1:** Sample information

**Breed**	**Line**	**Breed code**	**Platform**	# **Samples**	**Relation**
Red Jungle Fowl	Red Jungle Fowl	aviandiv_101	Aglilent 244K	4	unrelated (F0)
Exp.Layer brown	Compton_BrL	LineBrL	Aglilent 244K	5	unrelated (F0)
Exp. Layer white	Compton_6_sub1	Line6_1_	Aglilent 244K	5	unrelated (F0)
Exp. Layer white	Eastlansing_6_sub3	Line6_3_	Aglilent 244K	5	unrelated (F0)
Exp. Layer white	Compton-N	LineN	Aglilent 244K	5	unrelated (F0)
Exp. Layer white	Compton_15I	Line15I_5_	Aglilent 244K	5	unrelated (F0)
Exp. Layer white	Compton_7_sub2	Line7_2_	Aglilent 244K	5	unrelated (F0)
Com. Broiler	commercial broiler_A	ComBroilA	Aglilent 244K	2	unrelated (F0)
Com. Broiler	commercial broiler_B	ComBroilB	Aglilent 244K	3	unrelated (F0)
Com. Broiler	commercial broiler _C	ComBroilC	Aglilent 244K	4	unrelated (F0)
Exp. Broiler	broiler_M	BroilM	Aglilent 244K	8	unrelated (F0)
Com.Layer brown	commercial layer brown_1	ComBrownL1	Aglilent 244K	4	unrelated (F0)
Com.Layer brown	commercial layer brown_2	ComBrownL2	Aglilent 244K	4	unrelated (F0)
Com.Layer white	commercial layer white	ComWhiteL	Aglilent 244K	2	unrelated (F0)
Silkie	Silkie	Silkie	Aglilent 244K	3	unrelated (F0)
total				64	

### aCGH data analysis and CNV calling

Unamplified genomic DNA (1 μg) was labeled with Cy3 (test samples) or Cy5 (reference sample). The Agilent Oligonucleotide Array-based CGH for genomic DNA Analysis protocol (v4.0:2006) was used for the labeling of the DNA, Hybridizations, washings, and scanning of the arrays. Self-self control hybridizations were performed by labeling the reference sample with Cy3 and Cy5. Fluorescence intensities ratios were extracted using Agilent Feature Extraction software (Agilent Technologies). Log2ratio profiles were then normalized using aCGH-Spline to remove dye biases and reduce experimental noise [33]. CNV detection was performed using a modified version of CNVfinder [32], where we optimized empirically significance thresholds using replicate self-self hybridizations. The CNVRs were obtained by merging overlapping CNVs according to similar criteria as described previously [21,34].

### Quantitative RT-PCR

Relative abundance of DNA copy number for candidate CNVs was quantified by TaqMan quantitative PCR using an adapted method previously described [35].

Primers and probes for *TNFSF13B* and ovotranferrin were designed using Primer Express (Applied Biosystems) (Additional file 3). For the PCR we used 1× real-time PCR mix, *TNFSF13B* forward primer (0.4 μM), and *TNFSR13B* reverse primer (0.4 μM), *ovo* forward primer (0.4 μM), and *ovo* reverse primer (0.4 μM), *TNFSF13B* FAM probe (0.2 μM), *ovo* VIC probe (0.2 μM), bovine serum albumin (10 μg per reaction). The PCR was performed using the TaqMan fast universal PCR master mix reagents (Applied Biosystems, Warrington, UK). Amplification and detection of specific products was performed using the Applied Biosystems 7500 Fast Real-Time PCR System with the following thermocycling parameters: 50°C for 2 min, 95°C for 10 min, followed by 40 cycles of 94°C (15 sec) and 60°C (1 min). Results are expressed in terms of the threshold cycle value (*C*_t_), the cycle at which the change in the reporter dye passes a significance threshold (Δ*R*_*n*_).

To account for variation in sampling and DNA preparation, the *C*_t_ values for *TNFSF13B*-specific product for each sample were normalized using the *C*_t_ value of the ovotransferrin product for the same sample. Normalized *C*_t_ values were calculated using the formula *C*_t_ + (*N*_t_' − *C*_t_') × *S*/*S*', where *N*_t_' is the mean *C*_t_ for ovotransferrin among all samples, *C*_t_' is the mean *C*_t_ for ovotransferrin in the sample and *S* and *S*' are the slopes of the regressions of the standard plots for the test *TNFSF13B* and ovotransferrin, respectively. This effectively achieves interpolations on the standard plots to obtain the *TNFSF13B C*_t_ values that would have been obtained had all samples had the same (mean) amount of ovotransferrin DNA.

Additional validation was performed using a quantitative PCR approach, as described by Weksberg et al. [36], to investigate the difference CNVs. Copy number was determined for 12 markers in 12 different CNVs. Primer3 webtool http://frodo.wi.mit.edu/primer3/ was used to design primers for qPCR validation. Amplicon length was limited between (50 bp – 100 bp) and regions with GC percentage between 30% and 60% were included, while avoiding runs of identical nucleotides. All other settings were left at their default. Details of the qPCR primers can be found in Additional file 4: Table S5. qPCR experiments were conducted using MESA Blue qPCR MasterMix Plus for SYBR Assay Low ROX from Eurogentec, this 2x reaction buffer was used in a total reaction volume of 12.5 μl. All reactions were amplified on 7500 Real Time PCR system (Applied Biosystems group). The copy number differences were determined by using a standard ∆Ct method that compares the mean Ct value of the target CNV fragments, determined from different input concentrations, compared to the mean Ct value of reference sample (UCD001).

### Functional gene annotation

Functional gene annotation is performed in DAVID (gene Functional Classification Tool, DAVID Bioinformatics Resources 6.7, NIAID/NIH at http://david.abcc.ncifcrf.gov[26]. Ensemble gene ids within CNVR were collected (Additional file 5) and used as the input file for DAVID. The EASE score and the modified Fisher Exact P-Value were given where the smaller, the more enriched. Cut off P-Value within this study was E10^-4^.

## Abbreviations

CNV: Copy number variation; CNVR: Copy number variation region; SNP: Single nucleotide polymorphism; RT-PCR: Reverse transcriptase polymerase chain reaction; aCGH: Array comparative genomic hybridization; GGA: *Gallus* gallus; FM: Fibromelanosis; MD: Marek’s disease; LL: Lymphoid leukosis.

## Competing interests

The authors have declared that no competing interests exist.

## Authors’ contributions

RPMAC, MAMG conceived the study and prepared the manuscript. TF and RR performed the statistical analysis. MSF and PK performed analysis of the experimental lines including validation. SS performed the validation in the Silkie breed. HHC provided DNA of the reference animal and one of the experimental lines. MSF, RR, HHC, PK, SS contributed advice and data on biological issues, provided analytical support and contributed to understanding the data. All authors read and approved the final manuscript.

## Supplementary Material

Additional file 1 **All CNVs detected within the 64 chicken using aCGH against the Red Jungle Fowl animal used for deriving the whole genome sequence.** The first column indicated the CNV number whereas column 2 indicates the CNVR number of this CNV. Further columns indicate start and end position of the CNV on a particular chromosome followed by the overall occurrence of this CNV in the 64 animals used. Detailed CNV occurrence per line is given in the following columns. The last 4 columns give a literature review of overlapping chicken CNV found by others including the inter-specific CNVs detected in other avian species. Click here for file

Additional file 2 **Presents the specific CNVs either fixed or variable within a line or over lines.** Yellow blocks indicate fixed CNV within a line, green blocks represent fixed CNVs in a certain group of lines whereas red blocks represent specific CNV fixed within one line. Click here for file

Additional file 3 **Oligonucleotide primers and probes used in real-time PCR of *TNFR13B.*** Click here for file

Additional file 4 Marker information for the validation of 12 CNVs by real time PCR. Click here for file

Additional file 5 **Genes completely or partial overlapping with the CNVRs.** For every CNVR the genes are reported with completely or partial including Ensembl gene id with start en end of this gene. In the last column potential gene name is given (according to Ensembl). Click here for file

## Supporting data

The aCGH data from this study is submitted to the NCBI Gene Expression omnibus (http://www.ncbi.nlm.nih.gov/geo) under accession no GSE19866. The following supporting data are available with the online version of this paper.
