# Supplementary material for: Large scale variation in DNA copy number in chicken breeds
Source: BMC Genomics. 2013 Jun 13;14:398. doi: 10.1186/1471-2164-14-398 (PMC3751642; doi:10.1186/1471-2164-14-398)
Supplement: Additional file 3 — Oligonucleotide primers and probes used in real-time PCR of TNFR13B. [file 1471-2164-14-398-S3.docx]

**Additional file 3:   Oligonucleotide primers and probes used in real-time PCR of *TNFR13B* .**

| **Target Sequence** | **Primer/Probe Name** | **Primer Sequence (5’ – 3’)** |  |
| --- | --- | --- | --- |
| Ch. ovotransferrin gene  Ch. *TNFR13B* | Ovo forward | CACTGCCACTGGGCTCTGT |  |
|  | Ovo reverse | GCAATGGCAATAAACCTCCAA |  |
|  | *Ovo probe*  *TNFR13B-Forward*  *TNFR13B-Reverse*  *TNFR13-Probe* | AGTCTGGAGAAGTCTGTGCAGCCTCCA  (5’ VIC label, 3’ TAMRA label)  ------------------------------------------------------------------------  TTGGTGATGATCTCAGCTTGGT  AGGCGTGAAGGCACCAACGTA  TCGTTGCATCCAAAATATGCCACAGTCTT  (5’ FAM label, 3’ TAMRA label) |  |
